# S11-1: How to reach women in difficult life situations and encourage their participation in physical activity: Insights from urban and rural areas

**DOI:** 10.1093/eurpub/ckae114.246

**Published:** 2024-09-26

**Authors:** Maike Till, Karim Abu-Omar, Heiko Ziemainz

**Affiliations:** Friedrich-Alexander-Universität Erlangen-Nürnberg, Germany, Department for Sport Science and Sport; Friedrich-Alexander-Universität Erlangen-Nürnberg, Germany, Department for Sport Science and Sport; Friedrich-Alexander-Universität Erlangen-Nürnberg, Germany, Department for Sport Science and Sport

## Abstract

**Purpose:**

The community-based participatory research project “BIG” aims to empower socially disadvantaged women (e.g. single-mothers, unemployed women) to engage in physical activity (PA) and exercise. These women face barriers to exercise, such as high registration fees, limited childcare options, and culturally insensitive programs. This study explored various approaches to reaching these women and compared strategies between rural and urban areas.

**Methods:**

Over the last three years, two cities and three rural districts in Bavaria, Germany, have adopted the BIG project, previously implemented in 17 cities. As part of the intervention-study, project coordinators participated in annual individual interviews with a specific focus on reaching women. A total of 11 interviews were transcribed and analyzed using an inductive qualitative content-analysis, assisted by MAXQDA software, to address the research objective.

**Results:**

Preliminary results suggest that women facing difficult life situations (e.g., being unemployed, single mothers or from a different cultural background) are more readily reached in urban settings compared to rural areas. This can be attributed to the better-equipped structures and networks present in urban areas. Cities usually have specific meeting points, such as women's centers, support centers for immigrants and asylum seekers, or integration officers. In rural areas, the engagement of individual volunteers is crucial, especially leaning on the efforts of pivotal figures within each community. These individuals contribute their networks and expertise to support these initiatives, having earned the trust of the citizens. While individual 'champions' are influential in both rural and urban areas, their efforts and the trust they establish play a more significant role in rural areas.

**Conclusions:**

The study results highlight the necessity for distinct approaches, particularly in rural areas. Subsequent initiatives should prioritize devising methods to implement projects in these regions and employ strategies to reach women facing difficult life situations, especially those residing away from existing services.

**Support/Funding Source:**

This study was funded by the GKV-Bündnis for health.

